# Association between Anxiety, Depressive Symptoms, and Quality of Life in Patients Undergoing Diagnostic Flexible Video Bronchoscopy

**DOI:** 10.3390/ijerph181910374

**Published:** 2021-10-01

**Authors:** Beata Brajer-Luftmann, Marcin Mardas, Marta Stelmach-Mardas, Dorota Lojko, Halina Batura-Gabryel, Tomasz Piorunek

**Affiliations:** 1 Department of Pulmonology, Allergology and Pulmonary Oncology, Poznan University of Medical Sciences, Szamarzewskiego 84 Street, 60-569 Poznan, Poland; halinagabryel@wp.pl (H.B.-G.); t_piorun@op.pl (T.P.); 2 Department of Oncology, Poznan University of Medical Sciences, Szamarzewskiego 84 Street, 61-569 Poznan, Poland; marcin.mardas@ump.edu.pl; 3 Department of Treatment of Obesity, Metabolic Disorders and Clinical Dietetics, Poznan University of Medical Sciences, Szamarzewskiego 84 Street, 61-569 Poznan, Poland; stelmach@ump.edu.pl; 4 Department of Adult Psychiatry, Poznan University of Medical Sciences, Szpitalna 27/33 Street, 61-701 Poznan, Poland; lojko@ump.edu.pl

**Keywords:** bronchoscopy, STAI, BDI-II, WHOQOL

## Abstract

Bronchoscopy is one of the basic invasive procedures in pulmonology accompanied by patients’ anxiety. This study aimed to find an association between predictors of state anxiety/depression and patient’s quality of life (QOL) with pulmonary symptoms undergoing diagnostic flexible video bronchoscopy (FVB). A total of 125 adult patients before FVB were included in a prospective observational study. The quality of life (QOL) was assessed by WHOQOL-BREF questionnaire, the depression possibility by the Beck’s Depression Inventory-II (BDI-II), and the anxiety level by Spielberger’s State-Trait Anxiety Inventory (STAI-S; STAI-T). Results show that the older patients and patients with more comorbidities showed a significantly higher anxiety level. The previous FVB under deep sedation significantly reduced state anxiety. A significantly positive association was found between the STAI score and total BDI-II score. More severe symptoms of anxiety were especially related to lower QOL (physical health, psychological and environmental domains) in patients. Statistically higher trait anxiety in lower social QOL domain scores was observed. Our findings show that high state and trait anxiety were associated with higher depression scores and lower quality of life in the elderly. It seems that the elderly and patients at risk of depression development require more attention in the clinical setting to minimize the anxiety accompanying the bronchoscopy.

## 1. Introduction

Flexible video bronchoscopy (FVB) is one of the safe and minimally invasive diagnostic and therapeutic procedures in pulmonology, although it may cause, e.g., breathing difficulties, cough laryngospasm, airway obstruction, respiratory depression, hoarseness, hemoptysis, fever, or loss of conscious during the procedure [1,2,3]. Although professionals usually explain the side effects of the medical procedure, the decision to undergo an FVB may create anxiety in patients [4,5], which is defined as a complex medical situation characterized by an increased surveillance in situations of uncertain danger or potential threats to the integrity of the organism [6,7]. It is worth mentioning that different types of anxiety disorders may accompany depressive symptoms [8,9] or patients suffering from depression may experience anxiety and any of these disorders can coexist with somatic diseases [7,8,10,11]. They are found even more significantly frequently in patients affected by pulmonary disease (e.g., asthma, chronic obstructive pulmonary disease (COPD), cystic fibrosis (CF), obstructive sleep apnea syndrome (OSA)) than in the general population [11,12,13,14]. It is far more important as pulmonary symptoms such as dyspnea, chronic cough, and hemoptysis may directly decrease the patient’s quality of life (QOL) [14,15], being one of the symptoms preceding the onset of depressive and/or anxiety disorders [16,17,18]. Looking deeper into intermediated, psychological factors influencing QOL of patients suffering from pulmonary disorders is a straightforward part of the health policy, as an interdisciplinary approach applied to patients can improve treatment outcomes.

This study aimed to find an association between predictors of state anxiety/depression, and patient’s quality of life (QOL) with pulmonary symptoms undergoing diagnostic FVB.

## 2. Materials and Methods

### 2.1. Study Design

This was a prospective observational study. Patients were recruited between January 2020 and March 2020 at the Department of Pulmonology, Allergology, and Pulmonary Oncology, Poznan University of Medical Sciences (Poland). All included patients underwent routine FVB for diagnostic purposes in the Bronchoscopy Laboratory. This laboratory is the university unit that follows all procedures related to good laboratory and diagnostic practice. All procedures were collected in the institution’s endoscopy documentation system (ENDOBASE 13.0, Olympus Corporation. 2013, Hamburg, Germany).

The study protocol was approved by the Bioethical Committee at Poznan University of Medical Science (No: 1077/19). All enrolled patients provided written informed consent. The study was conducted in accordance with the Helsinki Declaration.

### 2.2. Study Population

**Figure 1:**
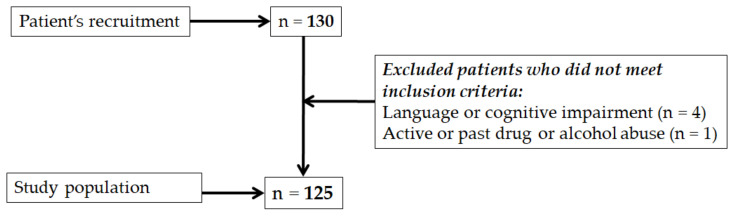
The patient sample selection.

The convenience sampling method was used to include 125 adult patients into the study according to the following criteria: age over 18 and diagnostic indication of FVB. The exclusion criteria were as follows: neurological disorders, dementia, or language or cognitive impairment that influences their ability to understand and fill out the questionnaire. Active or past drug or alcohol abuse, legal incompetence, and limited legal competence were additional exclusion criteria. The study flow chart is presented in Figure 1.

### 2.3. Patients and Methods

Patients were examined with the use of FVB in the morning. Patients’ data were collected using a structured questionnaire administered by a respiratory physician that underwent uniform training to conduct standardized interviews and collect the data. The questionnaire was designed to collect patients’ information regarding socio-demographic factors such as demographics, body mass index (BMI), educational level, marital and living status, smoking status, alcohol consumption, comorbidities, indication to FVB, past bronchoscopy under local or deep anesthesia, and admission to inpatient or outpatient clinic.

Anxiety was evaluated by the Spielberg Trait-State Anxiety Inventory (STAI) [19,20]. The Polish 2011 STAI version provided by the copyright owner (Psychological Test Laboratory of the Polish Psychological Association, Warsaw 2011, Poland) was used [21]. The STAI is a questionnaire consisting of two scales: the X-1 scale is used to test state anxiety, and the X-2 scale to test trait anxiety. Each of them consists of 20 items. The patient’s task was to respond questions by indicating the number on a scale of 1–4 that in the best way describes his subjective feelings via self-evaluation questionnaire. The state anxiety score ranges from a 20 to 80 raw points and can be transformed into ranges (stens). This study used raw results. A low score indicates no or little anxiety while a higher score describes a higher level of anxiety. All items of the state and trait anxiety scale were found to belong to one unidimensional scale.

An assessment of the possibility of depressive disorders in analyzed patients occurrence one week before the FVB was done with the use of the Beck Depression Inventory II (BDI-II) questionnaires [22]. This is a self-assessment questionnaire used to screen for symptoms of depression consisting of 21-items, where patients are asked about the particular symptom that bothered them. The symptoms were rated on a 4-point scale ranging from “not all” to “severely”. The patient could achieve between 0 and 63 points. Total scores of 1–13 were considered no or minimal depression, 14–19 as mild mood disturbances, 20–28 as moderate depression, and 29–63 indicating severe depression [23].

World Health Organization Quality of Life-BREF (WHOQOL-BREF) was used to assess the QOL of patients before the FVB [24,25]. The WHOQOL-BREF consists of four domains (physical health, psychological health, social relationships, and environment) and two items concerning overall QOL and general health. The five-point Likert type ranging from 1 (not at all/never/very dissatisfied/very poor) to 5 (extremely/always/very satisfied/very good), and the time frame, i.e., the previous two weeks, are similar as in the WHOQOL-100. Domain scores were scaled in a positive direction (i.e., higher scores denote higher quality of life). The mean score of items within each domain were used to calculate the domain score [24].

FVB was performed by an experienced pulmonologist/bronchoscopist and assisted by a dedicated trained health-care professional team including the anesthesiologist and endoscopic and anesthesiologic nurses. The procedures were performed in accordance with the applicable guidelines, including indications and contraindications [2,26]. The examination was done in the supine position after intravenous premedication with fentanyl followed by sedation with propofol, which was administered as bolus injections until the Richmond Agitation-Sedation Scale of 3 was achieved [27,28]. Patients were monitored throughout the procedure with a continuous electrocardiograph (ECG), oxygen saturation, and repeated noninvasive blood pressure measurements.

### 2.4. Statistical Analysis

A population of 118 subjects was required to show differences with type I error stated as alpha 0.05 and with the power of 80%. The data were shown as mean and standard deviation. The normality of the distribution was checked by the Shapiro–Wilk test. The Mann–Whitney test was used to analyze the differences between variables. The data on STAI were classified according to the following cut-off levels to describe the severity of anxiety as mild (≤35 points), moderate (35–45 points), or severe (≥46 points) being considered as clinically significant. A Chi square test was used for frequency distribution in categorical data.

Correlations between Beck depression scale, patient’s quality of life, and STAI-state/STAI-trait were calculated. The following interpretation of the size of correlation coefficient was applied: 0.0–0.19 (0.0–−0.19) very weak positive (negative) correlation, 0.2–0.39 (−0.2–−0.39) weak positive (negative) correlation; 0.4–0.59 (−0.4–−0.59) moderate positive (negative) correlation, 0.6–0.79 (−0.6–−0.79) strong positive (negative) correlation, and 0.80–1.00 (−0.8–−1.0) very strong positive (negative) correlation.

A *p-*value < 0.05 defined statistically significant differences. All calculations were two-tailed and done with the use of Statistica 10 software (TIBICO Software INC., Palo Alto, CA, USA).

## 3. Results

### 3.1. Subject Characteristic

The majority of the study group was women. The study group consisted mainly of patients characterized by normal body weight or overweight. Most of them had secondary and higher education and come either from villages or small towns. In most patients, FVB was performed on an outpatient basis. More than half of patients had the following comorbidities: pulmonary disease (91.3%) and hypertension (34.8%). The Charlson Comorbidity Index (CCI) was calculated taking into account age and coexisting diseases such as COPD, CF, lung cancer, diabetes, kidney failure, chronic heart failure, and hematologic disorders. The baseline characteristics of the study group are presented in Table 1.

### 3.2. Depression and Anxiety Scores in Patients Undergoing Diagnostic FVB

As shown in Table 2, the mean BDI-II total score was 9.2 ± 8.6, STAI-S score was 41.9 ± 10.49, and STAI-T 41.7 ± 8.6. Most of the participants showed a lack of or minimal depressive symptoms, several percent of patients revealed mild depression, and only a few percent of them presented moderate and severe depression.

### 3.3. Association between Anxiety, Depression, Quality of Life, and the FVB Procedure

The significantly positive association was found between STAI-state score and total Beck score indicating interrelation between both measures in the study group of patients. More severe symptoms of anxiety were significantly related to lower QOL in patients, especially in physical health, psychological, and environmental domains (Table 3).

The used classification in STAI state score showed that patients characterized by the highest value of score (≥46) were significantly older, suffered from mild mood disturbances, and had the lowest QOL with the highest percentage of depression risk development. The results of STAI-trait score indicated that the patients who achieved higher scores had more comorbidities and lower QOL with highest percentage of depression risk development (Table 4).

Patients who had previously undergone FVB revealed statistically significantly lower experience both of state anxiety and trait anxiety (*p*-value 0.0007 and <0.0001, respectively). Similarly, the use of deep sedation during the previous FVB significantly reduced both the feeling of state anxiety and trait anxiety (*p*-value < 0.0001 and 0.0475, respectively). The state and the trait anxiety measured using STAI-S and STAI-T score were not influenced by the procedure itself, which was performed during hospitalization or as outpatient setting.

## 4. Discussion

The current study focused on an association between anxiety level, the possibility of depressive disorder occurrence, and QOL in patients undergoing diagnostic FVB. The simple tools of questionnaires for anxiety and depression assessment were used. The high state and trait anxiety were associated with age, high depression score, and low score in the WHOQLQ questionnaire. The current study results suggest that the elderly and patients with an increased risk of depression development require more attention in the clinical setting to minimize the anxiety accompanying the bronchoscopy. Thus, identifying the patient’s group as being at higher risk of psychosomatic disorders development beforehand is highly important. It may directly influence bronchoscopy performance, compliance, and simply patient satisfaction with the endoscopic procedure. We recommend this simply approach to establish anxiety rates in other endoscopic procedures as well.

The gold standard for assessing anxiety is the STAI questionnaire, which includes state and trait assessment [20,29]. The current study showed that elderly people present a higher STAI-state and also STAI-trait inventory scores. Overdiagnosis or misdiagnosis of anxiety at an older age may be related to the individual perception and the way of describing fear feeling by the elderly [30]. Nevertheless, previously published data confirmed anxiety disorders in the elderly [30,31]. These data are in line with Aljohaney’s results [4], which confirmed that elderly patients feel more anxious than younger patients before bronchoscopy [4]. However, the onset of anxiety disorders may occur in the younger population [32]. This thesis supports study results by Poi et al. [5] indicating that older patients are less likely to be fearful than young. It was suggested that the elderly are more likely to accept invasive procedures with a better degree of stoicism and self-control. However, authors [5] did not use standardized questionnaires for anxiety assessment. Sargin et al. [33] assessed the patient anxiety level with the use of the Beck Anxiety Inventory scale and did not find an association between anxiety level and age of study patients. It seems that in the current study, the more pronounced anxiety related to FVB in the elderly may be explained by limited understanding of the procedure and by the fear of complications itself or even by the life-threatening consequences of the procedure, taking into account the advanced age of patients. Of course, different methods, e.g., questionnaire applied to assess the anxiety level in the group of study patients, could strongly influence obtained results [4,5,30,33,34,35].

Among significant factors related to feeling fear or anxiety before FVB by the patient were listed: gender, BMI, education level, and place of living [4,5,33]. One of the first observational studies conducted by Poi et al. [5] showed that females were significantly more fearful than males before bronchoscopy. Sargin et al. [33] achieved similar results connected to gender, where higher anxiety scores were exhibited in women than in men before an endoscopic procedure. These studies’ results are in line with the population data, confirming a more frequent occurrence of fear feeling in women [36]. According to the literature [37,38,39], higher anxiety level occurs in obese/overweight people in comparison to normal weight individuals. It seems that obese people do not cope properly with the confrontation/perception by the environment [40]. Aljohaney [4] also confirmed that obesity is a significant predictor of high anxiety before endoscopy. Our study does not support these results, which can be related to the fact that the patients are informed before the procedure that the general anesthesia will be used and that they will not feel any discomfort or pain and sleep during the FVB. Additionally, apart from written information, the patient receives an oral explanation in a manner appropriate to the patient’s education level to better understand the method and meaning of the applied medical procedure. It seems that additional attention paid to mental health may significantly reduce the level of anxiety. It is known that depressive disorders coexist with pulmonary symptoms and diseases [41,42,43]. According to our data, the possibility of depression development may be associated with anxiety even before the diagnostic FVB. The link between BDI-II score and STAI-S and STAI-T before the FVB has not been previously evaluated. On the one hand, patients with more severe symptoms of depression experience greater situational anxiety related to the stress caused by the procedure itself. On the other hand, severe depressive symptoms may contribute to an increased level of anxiety related to the personality structure [44,45,46].

The obtained study results indicating higher anxiety level before FVB and lower QOL are not surprising. Some studies have already revealed that anxiety is related to a reduced QOL [18,47,48,49], also taking into account QOL in particular age categories [48] and specific diseases [50,51,52]. Most of them proved that the presence of the somatic disease entity (e.g., SAS, COPD, asthma) and older age decrease QOL, especially physical health, psychological, and social QOL domains [50,52,53]. In our study, the patients with the better QOL perception and health perception felt less state and trait anxiety. A similar relationship between physical, psychological, and environment domains and STAI-S was demonstrated. Additionally, the patients with better QOL in the four main domains, including the social domain, presented a lower perception of anxiety related to the personality structure. This finding highlights the impact of QOL on anxiety, which also concurs with many other studies [18,53,54,55,56].

The earlier studies showed that patients with chronic medical disorders (diabetes, pulmonary disease, heart disease, arthritis) present a high level of anxiety [11]. It must be highlighted that the feeling of fear may be connected with the diagnosis itself and the severity of the disease [9,13]. The current study revealed comorbidities in more than half of included patients. We applied the CCI in our study to better explain the link between comorbidities occurrence and risk of anxiety in the analyzed group of patients [57]. Interestingly, we did not observe CCI influence on the STAI-S and STAI-T. However, when we assessed the relationship between the CCI and the obtained STAI score, it turned out that a higher CCI may impact a higher level of trait anxiety. Surprisingly, these data may suggest that a greater number of comorbidities did not affect the state anxiety, which may be aggravated before an invasive medical procedure.

The previous studies’ results revealed that patients who had had a bronchoscopy in the past were less fearful and anxious than the first time [5,58]. The current study supports this observation. Our group of patients felt less state and trait anxious if they had had FVB in the past. Additionally, the feeling of state anxiety was decreased if FVB was performed under deep sedation. These data may indicate that performing FVB under short general anesthesia improves the patient’s comfort and reduces the procedure’s anxiety. While the previous bronchoscopy affects the feeling of situational anxiety, it has a much lower impact on the sense of trait anxiety.

### The Study Strength and Limitations

The main limitations and strengths of the present study are as follows: (1) it was a single-center study with relatively small sample size (unbalanced between men and women); however, all FVBs were performed in the same way under short general anesthesia. In the patients whose had had a bronchoscopy in the past, most of them had an identical procedure at our Bronchoscopy Laboratory. The anxiety experience related to the FVB could also be connected to the type of anesthesia used in the study. (2) It was a non-randomized study. (3) The number of patients included in the presented study was a relatively small sample size. Therefore, the obtained study results should be taken with the caution.

## 5. Conclusions

In conclusion, the results of our study reveal that older age, a high depression score, and a lower QOL are the main predictors of state anxiety before FVB. Additionally, the CCI influences an increased trait but not state anxiety feeling. Therefore, a routine anxiety assessment before a bronchoscopy seems necessary, especially in the elderly, in patients showing symptoms that may suggest depression and/or characterized by lower QOL. Indicating people with a higher level of anxiety among patients waiting for FVB would help select the group that may need more nonpharmacological interventions, e.g., longer conversation with person in which the forthcoming procedures are explained and questions are answered. Additionally, this selecting group of patients may need anxiolytics for premedication. Such a procedure can increase patients’ comfort and reduce the unpleasant sensations associated with performing bronchoscopy in both outpatients and hospitalized patients.

## Figures and Tables

**Table 1:** The baseline characteristics of the study group.

Analyzed Variable	Mean ± SD or %
Sex (% of men)	39%
Age (years)	54.2 ± 16.9
	Range: 19–87
BMI (kg/m^2^)	24.6 ± 4.8
	Range: 16.1–37.4
BMI (kg/m^2^) categories (%)	
<18.5 (Underweight)	10.6%
18.5–24.9 (Healthy weight)	42.6%
25.0–29.9 (Overweight)	32.8%
≥30.0 (Obesity)	14.0%
Level of education (%)	
Primary school	29.0%
High school	35.5%
University degree	35.5%
Place of living (%)	
Village	34.7%
Cities < 50,000 inhabitants	11.3%
Cities 50,000 ≤ inhabitants < 100,000	15.3%
Cities ≥ 100,000 inhabitants	38.7%
Inpatients/outpatients (%)	28.2/71.8%
Previous FVB (%)	
No	51%
Yes	49%
Previous endoscopy—sedation type (%)	
Local sedation	17%
Deep sedation	83%
Comorbidities (%)	55.6%
No of comorbidities	Mean within 2.5 (range: 1–10)
No of comorbidities	
Charlson Comorbidity Index for all patients	2.35 ± (2.39) (range 0–11)

Data shown as: mean ± SD or %. Abbreviations: BMI, body mass index; FVB, flexible video bronchoscopy.

**Table 2:** The prevalence of depression and anxiety symptoms among the study group.

Depression and Anxiety Scores	Mean ± SD; Range or %
BDI-II total score:	9.2 ± 8.6; 0 to 46
BDI-II (%):	
No or minimal depression	73.5%
Mild depression	16.5%
Moderate depression	6.7%
Severe depression	3.3%
STAI-S	
Score:	41.9 ± 10.4; 21 to 72
STAI-T	
Score:	41.7 ± 8.6; 21 to 70

Data shown as: mean ± SD or % (number). Abbreviations: BDI-II, Beck’s Depression Inventory II; STAI-S, Spielberg State Anxiety Inventory; STAI-T, Spielberg Trait Anxiety Inventory.

**Table 3:** Association between anxiety, depression, and quality of life in patients undergoing diagnostic FVB.

Analyzed Variable	STAI-State			STAI-Trait		
	*r*	*p-*Value	*r* ^2^	*r*	*p-*Value	*r* ^2^
	**BDI-II**					
Total score	0.39	<0.0001	0.14	0.65	<0.0001	0.46
No or minimal depression	−0.25	0.0072	0.13	−0.58	<0.0001	0.42
Mild depression	0.25	0.0072	0.07	0.49	<0.0001	0.18
Moderate depression	0.12	0.1851	0.03	0.18	0.0474	0.16
Severe depression	0.13	0.1739	0.02	0.29	0.0015	0.04
	**Quality of Life ^a^**					
Physical health (D1)	−0.43	<0.0001	0.26	−0.41	<0.0001	0.27
Psychological (D2)	−0.39	<0.0001	0.14	−0.52	<0.0001	0.21
Social relationship (D3)	−0.15	0.0966	0.03	−0.40	<0.0001	0.13
Environment (D4)	−0.25	0.0064	0.06	−0.47	<0.0001	0.19
Total QOL	−0.39	<0.0001	0.2	−0.57	<0.0001	0.38

^a^ WHOQLQ-BREF; Abbreviations: BDI-II, Beck’s Depression Inventory II; NS, not significant; QOL, quality of life; r, Spearman correlation coefficient; STAI-S, Spielberg State Anxiety Inventory; STAI-T, Spielberg Trait-Anxiety Inventory.

**Table 4:** The STAI-score classification, depression, and quality of life in patients undergoing diagnostic FVB.

Analyzed Parameters	STAI-S Classification				STAI-T Classification			
STAI Score (Points)	≤35	36–45	≥46	*p-*Value	≤35	36–45	≥46	*p-*Value
Sex (Female/Male)	18F/14M	33F/19M	21F/12M		11F/15M	40F/20M	21F/13M	
Age (years)	51.3 (18.6)	52.4 (15.5)	60.3 (16.7)	0.0432	47.8 (16.6)	54.6 (16.1)	60.8 (14.0)	0.0285
BMI (kg/m^2^)	23.76 (4.6)	24.74 (4.9)	25.32 (4.2)	0.4886	24.3 (5.0)	24.2 (4.6)	27.1 (4.9)	0.0675
CCI	1.56 (1.50)	2.6 (2.5)	2.7 (2.4)	0.1514	1.3 (1.1)	2.6 (2.5)	2.7 (2.6)	0.0405
**BDI-II**								
Total score	6.8 (6.1)	7.9 (6.3)	13.9 (10.0)	0.0009	2.9 (2.8)	7.6 (5.7)	17.2 (8.7)	<0.0001
**Quality of Life ^a^**								
Physical health (D1)	23.7 (2.8)	21.9 (2.6)	19.8 (3.5)	<0.0001	23.3 (3.4)	22.1 (2.7)	19.6 (3.0)	0.0006
Psychological (D2)	22.1 (2.8)	20.7 (2.9)	19.0 (4.1)	0.0011	23.1 (1.9)	20.8 (2.7)	18.6 (4.8)	<0.0001
Social relationship (D3)	11.7 (1.9)	10.8 (2.4)	11.0 (2.0)	0.1469	12.4 (1.6)	10.9 (2.3)	10.6 (2.3)	0.0015
Environment (D4)	31.2 (5.5)	29.6 (6.2)	28.1 (4.9)	0.0242	33.9 (4.5)	29.2 (5.4)	27.8 (6.4)	0.0002
Total QOL	95.5 (11.2)	89.0 (11.6)	83.3 (11.4)	<0.0001	98.8 (11.6)	89.5 (10.3)	81.8 (11.2)	<0.0001
**Depression risk %**	15.6	29.6	54.5	0.0016	3.8	20.0	76.5	<0.0001

^a^ WHOQLQ-BREF, Abbreviations: BDI-II, Beck’s Depression Inventory; BMI, body mass index; CCI, Charlson Comorbidity Index; NS, not significant; STAI-S, Spielberg State Anxiety Inventory; STAI-T, Spielberg Trait-Anxiety Inventory.

## Data Availability

To get access to secondary data, please contact correspondence author.
